# Epidemiology of foodborne bongkrekic acid poisoning outbreaks in China, 2010 to 2020

**DOI:** 10.1371/journal.pone.0279957

**Published:** 2023-01-11

**Authors:** Hexiang Zhang, Yunchang Guo, Lili Chen, Zhitao Liu, Junhua Liang, Mengmeng Shi, Fei Gao, Yunqi Song, Jiang Chen, Ping Fu

**Affiliations:** 1 Zhejiang Provincial Center for Disease Control and Prevention, Hangzhou, Zhejiang, China; 2 National Health Commission Key Laboratory of Food Safety Risk Assessment, China National Center for Food Safety Risk Assessment, Beijing, China; 3 Yunnan Provincial Center for Disease Control and Prevention, Kunming, Yunnan, China; 4 Guangdong Provincial Center for Disease Control and Prevention, Guangzhou, Guangdong, China; 5 Guangxi Center for Disease Prevention and Control, Nanning, Guangxi Zhuang Autonomous Region, China; 6 Heilongjiang Provincial Center for Disease Control and Prevention, Haerbin, Heilongjiang, China; 7 Liaoning Provincial Center for Disease Control and Prevention, Shenyang, Liaoning, China; Beni Suef University Faculty of Veterinary Medicine, EGYPT

## Abstract

Foodborne bongkrekic acid (BA) poisoning is a fatal foodborne disease in China. From 2010–2020, a total of 19 BA poisoning outbreaks were reported to the China National Foodborne Disease Outbreak Surveillance System. These outbreaks involved 146 illnesses, 139 hospitalizations, and 43 deaths, with a case-fatality rate of 29.5%. Approximately 73.3% of the outbreaks occurred in South and Southwest China. Homemade fermented corn flour products, tremella, and sweet potato flour and corn flour products (jelly) caused more early outbreaks, and novel vehicles (wet rice noodles and Auricularia auricula) were associated with later outbreaks in the study period. Outbreaks most frequently occurred at home (79.0%) and in restaurants (21.0%). The prohibition of traditional processed homemade fermented corn flour products and improvement in bongkrekic acid poisoning case identification and early treatment have resulted in a reduction in the case-fatality rate.

## 1. Introduction

Foodborne bongkrekic acid (BA) poisoning, a notifiable disease in China, leads to high fatality in food poisoning cases produced by the bacterium *Burkholderia gladioli* pathovar *cocovenenans* (*B*. *cocovenenans*). BA is a potent toxin, and studies on mice suggest that the LD50 of BA by intragastric administration was 3.16 mg/kg [1]. Doses as small as 1–1.5 mg can be fatal in humans [2]. Reported symptoms include gastrointestinal and neurological symptoms, and critically ill patients experience failure of multiple organs, such as the liver and kidney [3, 4]. BA has been implicated in various outbreaks of severe foodborne illness in China [5], Indonesia [3] and Mozambique [6], and mortality rates from past outbreaks are as high as 30%-100% [5–8]. Implicated foods in outbreaks of foodborne BA poisoning are usually associated with fermented cereal products, spoiled tremella fuciformis and potato products in China [5]; in Indonesia, an outbreak was attributed to a traditional food made of coconut pulp fermented by *Rhizopus oligosporum* [3], and more recently, an outbreak in Mozambique was due to drinking pombe, a traditional alcoholic beverage made from corn flour [6].

A total of 103 outbreaks caused by *B*. *cocovenenans* were reported from 1985–1994; these outbreaks resulted in 667 illnesses and 301 deaths, with a fatality rate of 45.1% [5]. Geng et al. reported a total of 16 Pseudomonas cocoa food poisoning outbreaks, with 153 cases of illness and 51 cases of death, and the case fatality rate was 33.3% from 2002 to 2016 [7]. *B*. *cocovenenans*, which is widely distributed in nature, can be isolated from soil, corn, soybean, and dry and semidried white fungus [9] and contaminates relevant products during the processing of raw materials. In 1992, the separation rate of *B*. *cocovenenans* in corn flour sold was as high as 59.6% in the Beijing area [10]. *B*. *cocovenenans* can grow in a wide temperature range and can survive at low temperatures. Long-term storage or improper storage of various food products, such as fermented cereals, white fungus and potato products, is conducive to *B*. *cocovenenans* growth and reproduction and produces toxins, thereby poisoning consumers. In Guangxi, Yunnan, Guizhou and other mountainous areas, rice and corn are staple foods, and improperly prepared rice and corn products in households have led to the region becoming a high incidence area of BA produced by B. cocovenenans [7].

The investigation of BA poisoning outbreaks provides useful information regarding implicated foods, clinical symptom formation and epidemiology, which could improve BA poisoning case identification and early treatment to reduce the case-fatality rate. This report summarizes BA poisonings reported to the China National Foodborne Disease Outbreak Surveillance System (CNFDOSS) from 2010–2020. We describe the characteristics of the outbreaks and affected patients in terms of clinical manifestations, geography, implicated foods, and laboratory results.

## 2. Materials and methods

### 2.1 Data sources

To improve foodborne disease surveillance, the China National Center for Food Safety Risk Assessment, previously known as a section of the Chinese Center for Disease Control and Prevention (CDC), established a comprehensive national foodborne disease surveillance platform in 2011 according to the Food Safety Law of the People’s Republic of China released of 2009. The China national foodborne disease surveillance includes the Foodborne Disease Surveillance and Reporting System, Foodborne Disease Outbreaks Surveillance System (CNFDOSS), and National Foodborne Diseases Molecular Tracing Network [11].

As a principal foodborne disease surveillance component, the CNFDOSS passively collected and summarized data on foodborne diseases from all levels of CDCs in the entire country. Before 2011, outbreaks were reported through a simple and inconvenient web-based system. By 2011, all provincial-, municipal-, and county-level CDCs were submitting outbreak reports through the Foodborne Disease Outbreaks Surveillance System with an updated form [12]. The year 2015 was selected as an important point because investigation reports also needed to be uploaded for each outbreak as S1 Data.

A foodborne disease outbreak is defined as an incident in which at least cases involve a similar illness resulting from the consumption of a common food. CDC investigators at all different levels need to enter the standardized outbreak data into CNFDOSS after conducting surveillance for foodborne disease outbreaks in China. The reporting criteria include outbreaks with at least two cases or at least one death. For each reported outbreak, CNFDOSS collects data on outbreak characteristics (e.g., dates, number of ill persons, locations, and etiologic agents), case patient characteristics (e.g., symptoms, health care seeking, and whether the illness resulted in death), the setting of food preparation, contributing factors, the implicated food vehicles, and outbreak conclusion [12, 13]. Each outbreak data point was carefully reviewed by higher authorities.

Although outbreaks were reported in our system during the period of 2010–2015, detailed investigation reports in the incident were lacking. To obtain more detailed information on BA poisoning during the period 2010–2015, we conducted a literature review of published reports of BA poisoning during 2010–2015 using 2 electronic databases (Chinese Medical Journal Network and CNKI). When outbreak information, such as the date and place of occurrence, was matched between public reports and CNFDOSS, the published data were used.

### 2.2 Case identification and characterization

A case of foodborne BA poisoning is confirmed based on clinical and epidemiologic evidence and a positive laboratory specimen that met at least 1 of the following criteria: (1) *B*. *cocovenenans* isolated from foods or clinical specimens and toxicity in animal (mice) tests or BA detected in strain cultures and (2) the detection of BA in serum, gastric aspirate, or implicated food. Clinical evidence was defined as epigastric discomfort, nausea, vomiting, mild diarrhea, dizziness, weakness, severe jaundice, hepatomegaly, subcutaneous hemorrhage, hematemesis, hematuria, oliguria, unconsciousness, restlessness, convulsions, convulsions, shock, and a generally lack of fever. Epidemiologic evidence includes implicated foods and seasonality [14]. The occurrence of BA was detected in clinical and food specimens at the CDC, which was responsible for outbreak investigation. If testing was not possible, the samples needed to be sent to the superior CDC with testing capabilities. The detection of BA and isolation of *B*. *cocovenenans* from foods and clinical specimens were performed according to the China National Food Safety Standards (GB/T 5009.18–2016 and GB 4789.29–2020) [15, 16].

## 3. Results

### 3.1 Outbreak characterizations

From 2010–2020, a total of 19 BA poisoning outbreaks resulting in 146 illnesses, 139 hospitalizations, and 43 deaths were confirmed by the CNFDOSS. The 11-year hospitalization rate was 95.2%, and the case-fatality rate was 29.5%. Incubation period data were available for 12 outbreaks; the shortest incubation period ranged from 0.3–16 hours, and the longest incubation period was 38 hours. In some outbreaks, the incubation period of the cases was shorter, the symptoms were severe, and even death occurred; in others, no such correlation was observed. Fifty-two cases reported sex and age, including 25 males and 27 females, with ages ranging from 2–70 years old.

### 3.2 Outbreak settings

Although 15 (79.0%) outbreaks occurred in households (i.e., the food was consumed in private homes; the raw materials, which were intended to be refrigerated, were purchased commercially and then processed and eaten after being stored at room temperature), 4 outbreaks were attributed to foods prepared and served in restaurants.

### 3.3 Implicated foods

Implicated foods can be roughly divided into four categories, including fermented corn flour products (ciba, fermented corn noodles, tang-yuan and cooked dough), wet rice noodles, auricularia auricula and tremella, and sweet potato flour and corn flour products (jelly). Of 19 outbreaks, 9 (47%) were attributed to homemade fermented corn flour products (including ciba, fermented corn noodles, tang-yuan, and cooked dough). Rice noodle products were implicated in 5 (26%) outbreaks. Three outbreaks involved auricularia auricula, which was identified as a new vehicle in 2018. The largest outbreak (n = 52 cases) during the study period was associated with tremella fuciformis. The sweet potato flour and corn flour product (jelly) was reported as the vehicle in 1 outbreak. The mortality rates caused by implicated food ranked from high to low as follows: 60% of deaths were caused by auricularia auricula, 51% were caused by homemade fermented corn flour products, 33%were caused by wet rice noodles and none were caused by tremella or sweet potato flour and corn flour products (jelly) (Table 1).

**Table 1:** Foodborne bongkrekic acid poisoning outbreaks, cases, hospitalizations, deaths, by food source and year, China, 2010–2020.

Implicated food source	year	Food source	Geographic Information (Province)	No. outbreaks	No. cases	No. hospitalizations	No. (%) deaths
Homemade fermented corn flour products*	2010–2015, 2018	Home-prepared foods	South China (Guangxi) Southwest (Yunnan, Guizhou) Northeast China (Liaoning, Heilongjiang)	9	65	63	33(50.8)
Wet rice noodles	2018, 2020	Commercial retail foods, Restaurant foods (include street stall)	South China (Guangdong)	5	21	17	7(33.3)
Auricularia auricula	2018, 2019,2020	Commercial retail foods	East China (Zhejiang) South China (Guangdong)	3	5	4	3(60)
Tremella	2012	Commercial retail foods	East China (Shandong)	1	52	52	0
Sweet potato flour and corn flour products (jelly)	2018	Home-prepared foods	Southwest China (Sichuan)	1	3	3	0

* Homemade fermented corn flour products include ciba, fermented corn noodles, tang-yuan and cooked dough.

Homemade fermented corn flour products, rice noodle products and Auricularia auricula were the main implicated foods in South China. Tremella and auricularia auricula were the most implicated foods involved in 3 outbreaks in East China. In Southwest and Northeast China, homemade fermented corn flour products were the main implicated foods.

**Fig 1:**
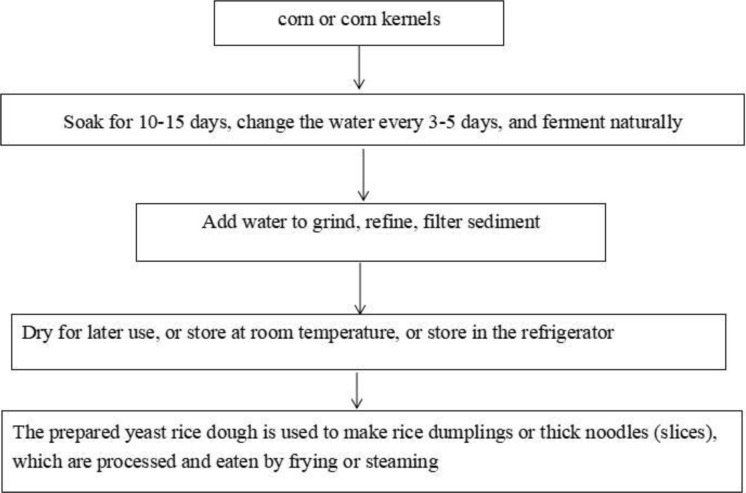
The production process of homemade fermented corn flour products.

Of the 19 BA poisoning outbreaks, 9 (47%) were caused by homemade fermented corn flour products. The preparation process is as follows. Briefly, corn or corn kernels are soaked in water and fermented naturally at a suitable temperature. When the corn kernels are soft and have a slightly sour taste, they are crushed, milled, filtered, and dried in the sun for storage. The wet dough is kneaded before serving to make ciba, fermented corn noodles, tang-yuan, and cooked dough (Fig 1).

### 3.4 Geographic information

**Fig 2:**
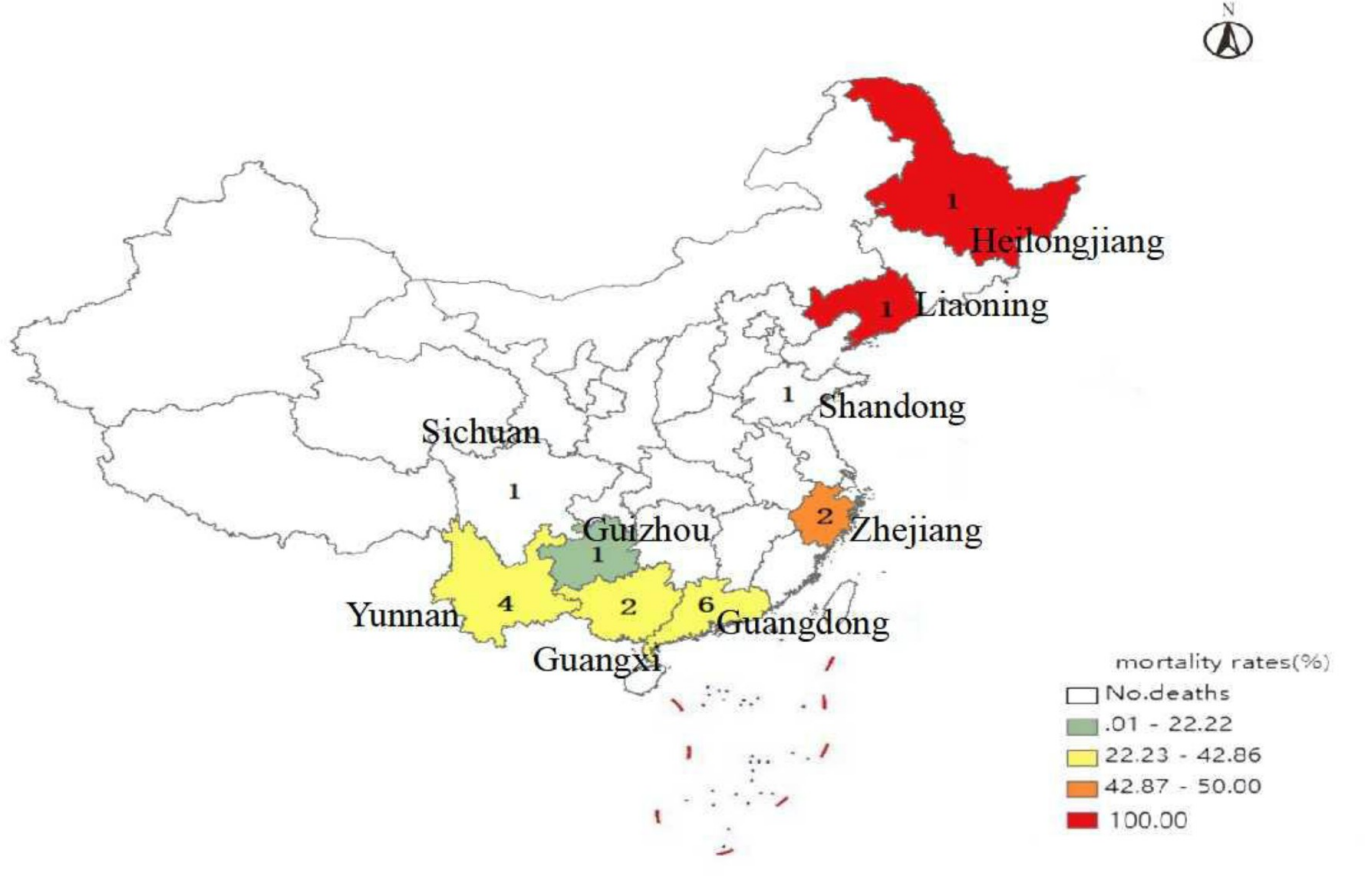
Total number and mortality rate of reported bongkrekic acid poisoning outbreaks by province, China, 2010–2020. Number given in each province indicates the total number of outbreaks; color boxes indicate the mortality rate.

BA poisoning outbreaks were reported from 9 provinces, with 146 cases documented in China during the study period. Most confirmed BA poisoning outbreaks (14, 73.3%) originated in South (Guangdong and Guangxi provinces) and Southwest China (Yunnan, Guizhou and Sichuan provinces). Reported cases in these places accounted for 52.7% (77/146) of the total number of cases (Table 2). The high number of cases recorded in the province was primarily due to a large family dinner-associated outbreak in Shandong that affected 52 persons (Table 2). A total of 43 deaths occurred in 9 provinces, with the highest case fatality rate of 100% occurring in Northeast China (Liaoning and Heilongjiang provinces) (Fig 2).

**Table 2:** Clinical manifestations of 134 cases with bongkrekic acid poisoning, China, 2010–2020.

Signs and Symptoms	All Cases (N = 134)
Vomiting	117(87.3%)
Nausea	86(64.2%)
Abdominal pain	46(34.3%)
Diarrhea	37(27.6%)
Vertigo	81(60.4%)
Weakness	66(49.3%)
Fever (37.0–37.5°C)	54(40.3%)
Loss of consciousness	39(29.1%)
Convulsions	6(4.5%)
Irritable	2(1.5%)
Chest tightness	2(1.5%)

* These are the symptoms the patient had before seeking medical attention.

### 3.5 Seasonality

**Fig 3:**
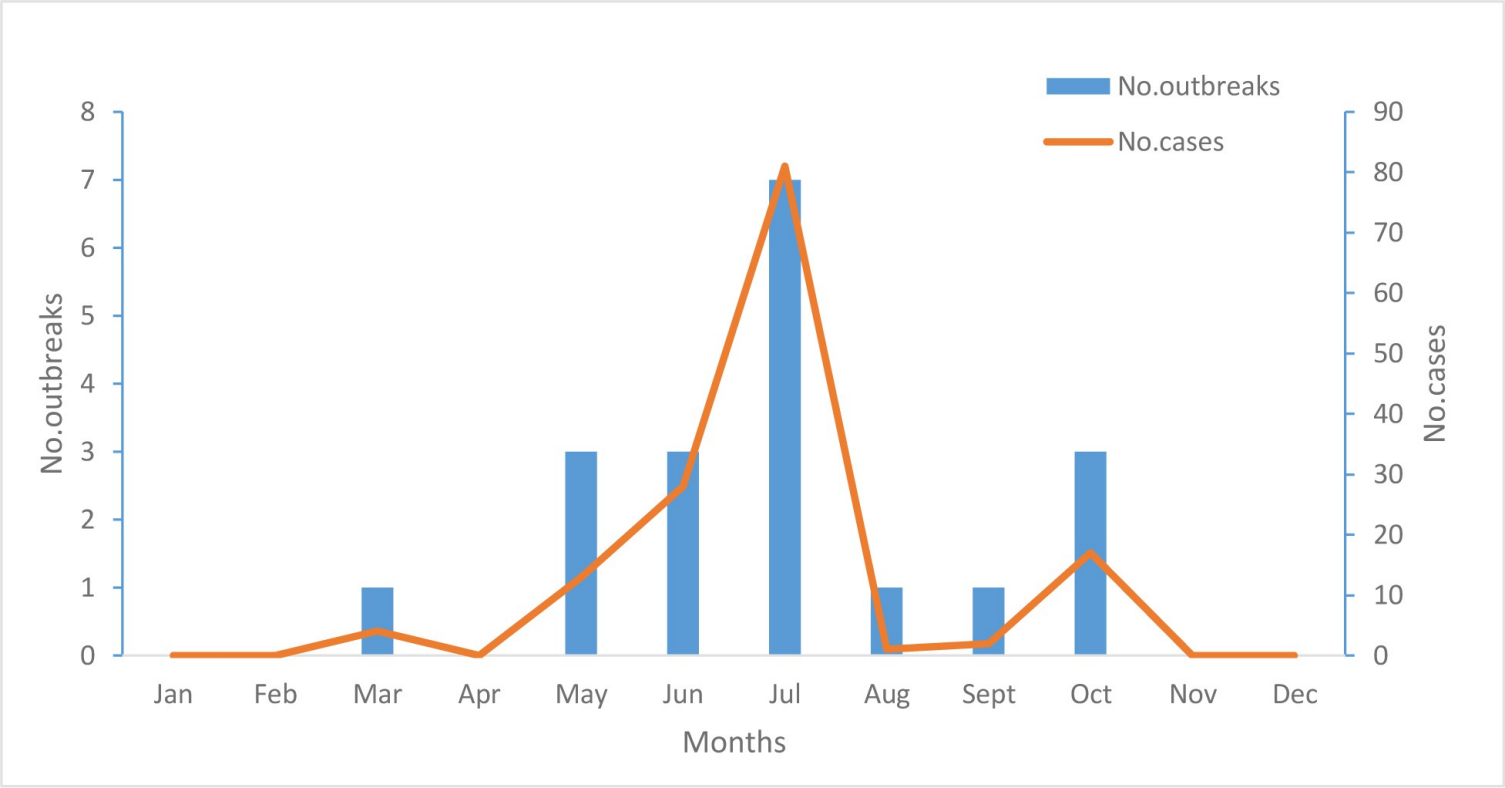
Reported bongkrekic acid poisoning outbreaks and outbreak-associated cases by seasons, China national foodborne diseases surveillance network, 2010–2020 (n = 19 outbreaks).

The majority of these outbreaks occurred during the warmer months, with 95% occurring from May to October; the peak month of occurrence was July (Fig 3).

### 3.6 Reported symptoms

The clinical manifestations of cases were analyzed, and cases with missing clinical manifestation data were excluded. Clinical manifestation data were available for 134 cases. Gastrointestinal symptoms, neurologic symptoms and severe liver or kidney function damage are the main characteristics of BA poisoning, which can lead to rapid death in a short time. Gastrointestinal symptoms included vomiting (n = 117, 87.3%), nausea (n = 86, 64.2%), abdominal pain (n = 46, 34.3%), and diarrhea (n = 37, 27.6%), and neurologic symptoms included vertigo (n = 81, 60.4%), weakness (n = 66, 49.3%), and a loss of consciousness (n = 39, 29.1%) (Table 3). Patients who died manifested decreased blood coagulation, acute liver function loss, myocardial damage, impaired renal function and other multiple organ failure, and a few fatalities also experienced high potassium, low blood pressure, shortness of breath, cyanosis of the lips and fingernails, and cardiac arrest. Fever symptoms mainly occurred in an outbreak involving 52 cases. Convulsions, irritability, and chest tightness were also noted in a few patients.

**Table 3:** Bongkrekic acid poisoning outbreaks, cases, and deaths by province in China, 2010–2020.

Province	No. (%) outbreaks	No. (%) cases	No. (%) deaths
Guangdong	6(31.6)	22(15.1)	8(36.4)
Yunnan	4(21.1)	36(24.8)	15(41.7)
Zhejiang	2(10.5)	4(2.7)	2(50.0)
Guangxi	2(10.5)	7(4.8)	3(42.9)
Liaoning	1(5.3)	4(15.1)	4(100.0)
Heilongjiang	1(5.3)	9(6.2)	9(100.0)
Guizhou	1(5.3)	9(6.2)	2(22.2)
Sichuan	1(5.3)	3(2.1)	0
Shandong	1(5.3)	52(35.6)	0
Total	19	146	43(29.5)

### 3.7 Occurrence of BA

BA was detected in food specimens, vomit, blood and urine samples from some patients. When food BA occurrence data were available, the median level of BA in food specimens was 1.5 (range: 0.78–330.2). The BA levels in blood samples were 335 μg/L (range: 70–879) for gravely ill patients or patients who died and 8.8 μg/L (range: 2.59–92) for mild and common cases (Table 4). Data regarding the consumption of suspicious foods were available for 13 patients, and doses as small as 15.4–154 μg could cause discomfort or illness, such as nausea, vomiting, dizziness, and fatigue.

**Table 4:** Results of laboratory analyses of bongkrekic acid in clinical specimens from some patients in China, 2010–2020.

Cases	Blood sample Median (range) (ug/L)	Urine sample (ug/L)^a^	Vomit specimens^a^
Mild cases and common cases	8.8 (2.59–92)	NA^b^	NA
Gravely ill patients and deaths	335 (70–879)	4.94 (<2.0–50.8)	3.16 mg/L, 2.15μg/kg, 37.9μg/kg

a If N < 5, the test results are directly listed.

b NA, not available.

## 4. Discussion

BA is a deadly toxin produced by *B*. *cocovenenans* that exists widely in nature and was isolated from wall layer soil, soybean storage, dried corn leaves, wild weeds, semidried white fungus and dried white fungus in the drying period [17, 18]. BA poisoning outbreaks have been reported in Asia and Africa [3, 5, 6] and are mainly associated with spoiled or fermented foods. As a rare and highly fatal foodborne disease, the 11-year characteristics BA poisoning outbreaks presented here represent a unique opportunity to describe the epidemiology, clinical features and laboratory results that might be useful for timely outbreak detection and improved diagnoses and treatment, which may prevent devastating consequences.

During the study period, an average of 1.7 confirmed outbreaks per year were reported. In contrast, an average of 10.3 outbreaks per year were reported during 1985–1994 [16]. The average case fatality rate caused by foodborne bongkrekic acid poisoning dramatically decreased from 45.13% during 1985–1994 to 29.5% during 2010–2020. From 1985–1995, BA poisoning outbreaks occurred mainly in rural and mountainous areas in China. In these areas, which are characterized by low living standards and limited food variety, residents often prepare their own fermented foods. Due to improper processing methods, fermented foods are usually contaminated with B. cocovenenans and its toxins, which leads to poisoning. Since BA poisoning was not included in the statistical analysis of foodborne diseases at that time, primary health institutions did not place sufficient focus on this type of food poisoning. The establishment of the rural three-level (county, township, village) medical prevention and health care network and the promulgation of the "Food Safety Law" in recent years has facilitated public health staff to conduct food safety education and disseminate scientific knowledge on the prevention of food poisoning in high-risk regions (including areas where cases occur, where homemade fermented foods are habitually eaten). At the same time, improvements in medical care have greatly reduced the incidence of disease and mortality. The decline in outbreak numbers and mortality could reflect the increased awareness of symptoms and consumer prevention education in high-risk regions, which leads to prompt medical attention. Changes in the characteristics of outbreaks reported to CNFDOSS during 2010–2020 highlight the successes and continued challenges of BA poisoning outbreaks.

Outbreak areas and implicated foods widely vary. South and Southwest China reported the most BA poisoning outbreaks, followed by East China and Northeast China, and no cases were reported elsewhere. BA poisoning outbreaks caused by fermented cornmeal products have occurred in places where people habitually prepare and eat fermented cornmeal products, such as South (Guangxi Province), Southwest, and Northeast China. Wet rice noodles are the main implicated foods in South China (Guangdong Province). These findings may suggest variable eating habits or food processing methods leading to the outbreak differences by region and implicated food.

Notably, fermented cereal products are home-prepared foods that cause outbreaks, whereas industrialized mass-produced cereal fermented products have not been found to cause poisoning. This difference suggests that fermented cereal products may be contaminated during production, such as the drying and storage process, resulting in a large amount of toxins. This finding reflects the importance of production to the occurrence of poisoning caused by cereal fermented products.

Two novel food vehicles, auricularia auricula and wet rice noodles, have been implicated in BA poisoning outbreaks described since 2018 in this review. Auricularia auricula was associated with three outbreaks. Wet rice noodle products were related to five outbreaks. Outbreaks that were linked to commercial wet rice noodle products are noteworthy because according to the epidemiological investigation reports, no peculiar smell, no color and character changes, and no taste abnormalities were observed in the process of cooking and eating the wet rice noodles. One study showed that spoilage was not evident because the food was not fermented and sodium dehydroacetate use was excessive [4].

BA is the most potent toxin produced by *B*. *cocovenenans*, and doses of 1.0–1.5 mg have been reported to cause death [2]. In this study, doses as small as 15.4–154 μg could cause discomfort or illness. A previous study showed that those who died from BA poisoning had a shorter time interval between eating suspicious food and disease onset than survivors [6]. In this study, the incubation period of the cases was shorter, the symptoms were severe, and even death occurred for some outbreaks; in others, no such correlation was observed. Overall, this study did not find a correlation between the incubation period and severity. In this study, we also report the occurrence of BA in suspicious food and biological samples, such as vomit, blood and urine samples. Gravely ill patients or patients who died generally had higher concentrations of BA in blood samples than patients with mild cases and common cases.

This study had a few limitations. Not all of the outbreak reports we examined included information on food preparation or handling practices that might have contributed to the outbreak. Similarly, incubation period data were limited or unavailable for several outbreaks, and the observed incubation period should be interpreted with caution. Second, reported foodborne BA poisoning outbreaks cannot represent all actual outbreaks. CNFDOSS, which is a passive monitoring system, relies on reporting by local hospitals. Smaller outbreaks with less severe disease presentation and cases that did not go to hospital, may have been unnoticed. Third, we did not analyze trends by year because of the few outbreaks that occurred each year. Despite substantial limitations in the current systems for CNFDOSS, the investigation of foodborne BA poisoning outbreaks provides an important opportunity to better understand the epidemiology of foodborne illness and develop preventive interventions.

## 5. Conclusions

We examined the epidemiology of foodborne bongkrekic acid poisoning outbreaks in China from 2010 to 2020. The results provided by our study indicated that measures could be taken to control foodborne bongkrekic acid poisoning outbreaks in China. The prohibition of traditional processed homemade fermented corn flour products and improvements in bongkrekic acid poisoning case identification and early treatment have resulted in a reduction in the case-fatality rate. Furthermore, specific publicity and education in outbreak areas are indispensable.

## Supporting information

S1 Data (XLSX)
